# Vaccines against malaria

**DOI:** 10.1098/rstb.2011.0091

**Published:** 2011-10-12

**Authors:** Adrian V. S. Hill

**Affiliations:** The Jenner Institute, University of Oxford, Old Road Campus Research Building, Oxford OX37DQ, UK

**Keywords:** vaccines, parasite, malaria

## Abstract

There is no licenced vaccine against any human parasitic disease and *Plasmodium falciparum* malaria, a major cause of infectious mortality, presents a great challenge to vaccine developers. This has led to the assessment of a wide variety of approaches to malaria vaccine design and development, assisted by the availability of a safe challenge model for small-scale efficacy testing of vaccine candidates. Malaria vaccine development has been at the forefront of assessing many new vaccine technologies including novel adjuvants, vectored prime-boost regimes and the concept of community vaccination to block malaria transmission. Most current vaccine candidates target a single stage of the parasite's life cycle and vaccines against the early pre-erythrocytic stages have shown most success. A protein in adjuvant vaccine, working through antibodies against sporozoites, and viral vector vaccines targeting the intracellular liver-stage parasite with cellular immunity show partial efficacy in humans, and the anti-sporozoite vaccine is currently in phase III trials. However, a more effective malaria vaccine suitable for widespread cost-effective deployment is likely to require a multi-component vaccine targeting more than one life cycle stage. The most attractive near-term approach to develop such a product is to combine existing partially effective pre-erythrocytic vaccine candidates.

## 1. Importance of malaria

**Figure 1.**
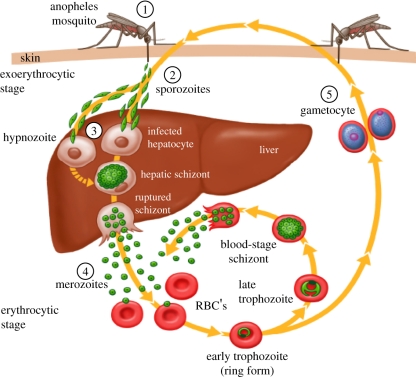
Life cycle of the malaria parasite illustrating the various stages that are relevant to vaccine design. These are (1) the anopheline mosquito vector, used in experimental protocols to immunize with irradiated sporozoites administered by mosquito bite; (2) the sporozoite, the target of several vaccines, including RTS,S; (3) the liver-stage, usually targeted by vectored vaccines; (4) the blood-stage, usually targeted by protein in adjuvant vaccine candidates. Merozoite antigens have been most often included in blood-stage vaccines; (5) the gametocyte which along with the ookinete, formed after fertilization in the mosquito midgut, is the source of parasite antigens used in sexual-stage transmission-blocking vaccines. Pre-erythrocytic vaccines, which target the sporozoite and the liver-stage parasite are intended to prevent infection as well as disease while blood-stage vaccines are intended to prevent clinical illness and death.

Malaria is the most important parasitic disease of humans and efforts to develop effective vaccines span more than six decades. Studies of inactivated sporozoite immunization reported in 1942 showed an apparently beneficial effect of combining induction of cellular and humoral immune responses against malaria of domestic fowl [1]. Around the same time, Freund was developing a powerful adjuvant that showed promising efficacy in malaria studies [2]. However, today, there is still no licenced vaccine against malaria or any other parasitic disease of humans and no deployed subunit vaccine for any parasitic disease of livestock [3,4]. Nonetheless, the continuing unacceptable impact of malaria morbidity and mortality, amounting to over 800 000 deaths and some 250 million clinical episodes annually [5] has led to a variety of sustained efforts to develop effective malaria vaccine candidates. In the last decade in particular, the development of vaccine candidates for malaria has accelerated considerably and one candidate has recently reached the stage of a large-scale phase III trial while other potentially complementary approaches are showing increasing promise. In this short review, I focus primarily on pre-erythrocytic vaccines (figure 1) as they have shown more promise than vaccines against other stages of the life cycle, but I also briefly survey a broader range of approaches.

## 2. Vaccines against parasites

Most of the vaccines that are available today belong to one of three categories—attenuated microbes, killed microbes or protein subunits. Attenuated viruses that protect against a cross-reactive pathogen originate with Jenner's use of a related poxvirus to prevent smallpox. Killed microbes were introduced a century later and several such vaccines, e.g. polio vaccine, are used widely. More recently, conjugate vaccines against encapsulated bacterial pathogens have been developed and have been hugely successful in reducing the incidence of some diseases. However, licenced subunit vaccines based on a protein are very few and these are particulate (composed of many copies of the expressed protein that assemble spontaneously into particles that are more readily process by immune cells), for example, the hepatitis B surface antigen and the human papilloma virus vaccines. Recombinant proteins that are not particulate have rarely become effective human vaccines. Parasitologists trying to develop vaccines can hardly ever safely grow and manufacture whole parasites in sufficient numbers to induce immunity, although recently this has been attempted for malaria [6]. Instead, a large number of antigens have been expressed, mainly as proteins and less often from vector systems to try to generate protective immunity [7]. This has proved difficult but possible in some murine models of malaria and this has encouraged many attempts at vaccination against *Plasmodium falciparum*, the major target species for malaria vaccine development. Parasite vaccines generally face the challenge of generating immunity with an immunogen that reflects only a tiny fraction (less than 1%) of the composition of the organism, a challenge that has been met only rarely in vaccinology. Encouragingly, in at least one system, vaccination of pigs and cattle against *Taenia*, vaccination with a single antigen has prevented cysticercosis causes by cestode parasites [8].

Although natural immunity to malaria develops in most residents of endemic areas, this generally takes some years of exposure and is imperfect. Extensive immunoepidemiological studies have provided limited insight into what the best antigens to include in a vaccine might be: natural immunity predominantly targets a wide variety of blood-stage antigens and no one antigen appears to be especially important in providing protection [9].

Several other difficulties have slowed progress. Most malaria antigens that have been selected as vaccine candidates are the targets of natural immunity and exhibit significant genetic polymorphism, and a key blood-stage antigen, *P. falciparum* erythrocyte membrane protein-1 (PfEMP1), even shows temporal switching of variant expression. There is substantial stage-specificity of antigen expression by *Plasmodium* parasites so that candidate vaccines for one stage of the life cycle (figure 1) are unlikely to impact on another stage. Finally, malaria vaccine developers are faced with target species *P. falciparum* and *Plasmodium vivax* that will not infect small animals or old world macaques, so excluding the most widely used animal models for straightforward vaccine evaluation. There are many other malaria parasites that infect these species but these differ substantially from human parasites.

## 3. A short history of malaria vaccine development

Modern malaria vaccine development stems from immunization studies of mice with irradiated sporozoites, conducted in the 1960s [10], and subsequent analyses of the mechanisms of immunity in this model [11]. Key challenge studies by Clyde in humans [12] demonstrated that a high level of protection could be induced in volunteers but required large numbers of bites by irradiated infectious mosquitoes. The identification of the circumsporozoite protein as the major component of the sporozoite coat led to the cloning and sequencing of this gene in the early 1980s and optimistic predictions that a sporozoite vaccine was within reach [13]. About this time, excellent progress was made in identifying and expressing a range of blood-stage antigens also raising hopes for a blood-stage vaccine. However, initial clinical trials revealed only modest immunogenicity of candidate antigens and no statistically significant efficacy on sporozoite challenge [14]. The emergence of a peptide-based candidate vaccine from Colombia, called SPf66, with apparent efficacy in new world monkeys and humans [15] generated enormous interest and controversy but eventually disappointment as successive, independent field efficacy trials in Africa and Asia failed to demonstrate protection. However, these studies with SPf66 led to the development of the field technologies used subsequently to evaluate other vaccines.

At about the same time, a new formulation of the *P. falciparum* CS protein, called RTS,S, in a novel adjuvant was showing exciting evidence of efficacy in sporozoite challenge studies [16] and this moved onto field testing in West Africa [17]. By then, the importance of cellular immunity in providing protection against the liver-stage of the parasite had been confirmed in animal models of irradiated sporozoite immunization [18]. This led to efforts to induce significant cellular immunity using a new approach—plasmid DNA immunization. The low potency of first generation DNA vaccines [19] led to the development of heterologous prime-boost immunization approaches with non-replicating viral vectors that showed some efficacy that could not be attributed to antibody-dependent immunity [20]. Over the last 10 years, the RTS,S candidate has led the way, showing efficacy in progressively younger subjects and in a variety of epidemiological settings culminating in an ongoing licensure trial [21]. Most recently, the old approach of whole parasite vaccination has been revived, aiming to induce considerably higher levels of efficacy than RTS,S despite substantial challenges in product development [6]. Blood-stage vaccine candidates continue to struggle with adjuvant formulations and limited immunogenicity while calls for efforts at malaria eradication have led to a revival of enthusiasm for the near-dormant field of transmission-blocking vaccine development [22].

## 4. A diversity of approaches

The difficulty of developing a highly effective malaria vaccine has led to the design and assessment of a very wide range of new approaches, arguably unparalleled in any other area of infectious disease vaccinology. This not only includes a diversity of approaches to control malaria infection and disease, as outlined in box 1, but also includes early assessment of a wide range of new vaccine technologies. The use of malaria as a testing ground for innovative new vaccine technologies has been helped by the ability to undertake ethical, small-scale challenge studies with either sporozoites delivered by mosquito bites or carefully qualified blood-stage inocula to assess vaccine efficacy [23]. Some of the firsts that the malaria vaccine community can point to are illustrated in box 2.

Box 1. **Twelve approaches to a malaria vaccine.**

1. Sporozoite subunit vaccination, especially with the CS protein: e.g. RTS,S in adjuvant.
2. Irradiated sporozoite or genetically attenuated sporozoite immunization either by mosquito bite or using injected purified sporozoites.
3. Immunization with DNA and/or viral vectors to induce T cells against the liver-stage parasites, or to target other life cycle stages.
4. Use of whole blood-stage malaria parasites as immunogens.
5. Use of protein in adjuvant vaccines to reduce the growth rate of blood-stage parasites.
6. Use of protein (or long peptide) in adjuvant vaccines to induce antibody-dependent cellular inhibition (ADCI) of blood-stage parasites.
7. Use of peptide-based vaccines, mainly against blood-stage parasites—e.g. SPf66, PEV3a.
8. Development of anti-disease vaccines based on parasite toxins—e.g. GPI-based.
9. Immunization with parasite adhesion ligands such as PfEMP1.
10. Use of parasite antigens, such as the Var2 protein, preferentially expressed in the placenta to prevent malaria in pregnancy.
11. Immunization with sexual stage parasite antigens as transmission-blocking vaccines.
12. Use of mosquito antigens as transmission-blocking vaccines.

Box 2. **Malaria vaccines: some pioneering advances.**

Development of novel adjuvant formulations inducing exceptional levels of antibody: e.g. AS01.

Recombinant particle development: e.g. RTS,S vaccine.

First reported clinical trial of DNA vaccination.

Large-scale testing of a peptide-based vaccine, SPf66.

Discovery and clinical development of prime-boost immunization with vectors.

Demonstration of T-cell-mediated protection with sub-unit vaccination.

Clinical assessment of recombinant virosome, multiple antigenic peptide and long peptide vaccines.

Development of the concept of community/transmission-blocking immunization, with sexual stage antigens.

Development of a pathogen challenge model widely used to guide vaccine development.

Development of a regulatory compliant process for whole parasite vaccine biomanufacture.

## 5. The leading vaccine candidate: RTS,S

The most effective malaria vaccine tested to date is RTS,S, a hybrid protein particle, formulated in a multi-component adjuvant named AS01. RTS,S results from a collaboration, commenced in the 1980s, between the Walter Reed Army Institute of Research in the USA and GSK Biologicals, then SmithKline Beecham [21]. Initial vaccine constructs of the tandem repeat region of the circumsporozoite protein, mainly copies of the four amino acid sequence NANP, showed very low-level efficacy [14] but expressing the central repeat (‘R’) fused to the C-terminal region known to contain T cell epitopes (hence ‘T’) fused in turn to the hepatitis B surface antigen (‘S’) yielded a yeast-expressed protein RTS [16]. However, to generate immunogenic particles, the RTS protein needed to be co-expressed with large amounts of the unfused S protein to yield RTS,S. RTS,S was tested with several adjuvant formulations in a key sporozoite challenge study in early 1996. The highest protective efficacy was observed in volunteers who received the vaccine with an adjuvant containing the immunostimulants, mono-phosphoryl lipid A (MPL, a toll-like receptor 4 agonist) and QS21 (a derivative of Quill A) [16]. When combined with this adjuvant, known as AS02, or the related adjuvant AS01, which contains liposomes, RTS,S has shown sterile efficacy of 30–50% across a series of sporozoite challenge studies in healthy volunteers. In addition, a further 20 per cent of those challenged show non-sterile efficacy manifest as a 2 day delay in the time to the appearance of parasites in the blood, an indication of substantial killing of parasites during or before the liver stage of the infection [24]. In sporozoite challenge studies if most, but not all, parasites are killed before the blood-stage of infection, the appearance of parasites at microscopically detectable levels will occur, but this will be delayed, and the extent of the delay allows the proportion of parasites killed to be estimated [25].

Immunological analysis has demonstrated the remarkable ability of this vaccine to induce a very high concentration of antibodies, often of hundreds of micrograms per millilitre, that target the conserved central repeat region of the circumsporozoite protein and in several, but not all, settings the level of these antibodies correlates with protection against infection or disease [26]. In contrast, T cell immunogenity is modest and suggestions that these low-level responses might also contribute to protection have, at least thus far, been unconvincing [27].

The level of efficacy achieved by RTS,S in challenge studies was a clear breakthrough for the field and has yet to be exceeded by any sub-unit vaccine candidate. RTS,S has progressed through a series of phase I and II clinical trials in several African countries, involving age de-escalation from adults to infants and various efficacy assessments. These provide clear evidence that in many different epidemiological settings, RTS,S can reduce the rate of acquisition of clinical malaria by 30–50% [28–31]. The endpoint most widely accepted as a semi-standardized efficacy measure is the reduction in clinical cases (or first episodes) of malaria during the first 12 months of follow-up, a measure alluded to in the Malaria Vaccine Technology Roadmap. By this measure, the only published results with the current AS01 adjuvant formulation is an efficacy of 39 per cent in East African children [31], a higher level than that observed previously with AS02.

RTS,S/AS01 is now in a very large phase III efficacy trial involving approximately 15 000 children at 11 sites in seven African countries, with the aim of licensure and deployment in about 2015. Some initial data from this trial, from evaluation in 5–17 month-old children rather than in younger infants, the primary vaccine target population, should be available by the end of 2011. This represents encouraging progress towards licensing of a first generation malaria vaccine. However, some important questions remain about the efficacy and utility of this vaccine for malaria control, not all of which will be answered by the current phase III trial. These include the level of efficacy against severe malaria, which could be higher than that against clinical malaria, the duration of protection provided by the vaccine, which was limited with the AS02 formulation both in phase IIa and phase IIb studies [29,32], and the cost-effectiveness and community acceptability of deploying a vaccine with limited efficacy.

## 6. Whole parasite vaccines

Partly as a response to the limited efficacy achieved by RTS,S and all other sub-unit vaccine candidates, a major effort has been made by a US biotech company, Sanaria, to develop a pre-erythrocytic vaccine comprising whole sporozoites [6]. The challenges facing this approach are considerable but the main driver has been the appreciation that irradiated sporozoites delivered by mosquito bite have induced very high levels of protective efficacy, exceeding 90 per cent, though the numbers of volunteers in these trials were small [33]. Irradiated sporozoites can invade liver cells and develop within them to produce defective schizonts. These express antigens that can induce a protective immune response but the defective schizonts cannot rupture to release the merozoites that would normally invade red blood cells and continue the infection. By analogy with animal models in which complete protection is readily obtainable, this efficacy is likely to be achieved through the activity of induced CD8^+^ T cells that clear infected human liver cells, but this remains to be demonstrated [11].

Because the delivery of about a thousand mosquito bites (the number required for high-level efficacy using the irradiated sporozoite approach) is impractical for a vaccine for public health use, Sanaria set out to establish a regulatory compliant manufacturing process that involved aseptic dissection of parasites from thousands of mosquito salivary glands [6]. The next step was to purify these parasites, irradiate them and then cryopreserve them in liquid nitrogen because the parasites lose viability at higher temperatures and they must remain viable to induce protection. Remarkably, both of these challenges appear to have been largely overcome and phase I/IIa clinical trials of this vaccine were undertaken in 2010. However, significant efficacy has yet to be reported from these studies and it remains unclear whether a needle and a syringe can substitute for a mosquito and its salivary gland fluids in generating adequate immunogenicity and efficacy in humans.

Even if high-level efficacy can be achieved using this approach, the challenges of cost of manufacture and distribution of parasite vials in liquid nitrogen tanks in developing countries suggest that efficacy of this approach will need to be considerable higher than that of other malaria vaccines, and possibly other control methods, for it to be deployed widely.

In parallel with efforts to develop irradiated sporozoite vaccines, considerable progress has been made in developing genetically attenuated parasites that are incapable of progressing beyond the liver-stage owing to loss of key gene(s) [34]. These parasites would not require irradiation to be used as a vaccine and might be more efficacious than irradiated parasites if they were able to progress to a later stage of liver-stage development than the former [34]. However, the lack of irradiation raises concerns over safety and ensuring a complete absence of break-through infections with such a vaccine might be difficult, even if more than one mutation is introduced. In addition, a delivery modality that retains the immunogenicity of mosquito-delivered vaccines will be required, as for the Sanaria approach. Further evidence of the potency of whole parasites for inducing immunity in humans has been provided by the demonstration of sterile protection induced by small numbers of infectious mosquito bites, about 45 non-irradiated mosquitoes in total, if these are administered with the anti-malarial drug chloroquine [35].

A further extension of the whole parasite approach has been championed by Australian researchers who have demonstrated the potential efficacy of blood-stage whole parasite vaccines in both animals and humans [36]. In a clinical trial, administration of repeated, very low doses of blood-stage parasites induced immunity to a subsequent challenge and this appeared to work in the absence of induced antibodies [37]. Efforts to develop this approach further are ongoing but the major question is whether a means of growing large numbers of parasites in blood or a blood-substitute can be developed which will be acceptable to regulatory authorities, given the tiny risk of unknown infections in any donated human blood.

## 7. Vectored vaccines

The third major approach to inducing pre-erythrocytic immunity has been to employ vectored vaccines, aiming mainly to induce cellular immunity against the liver-stage of *P. falciparum*. Irradiated sporozoite-induced immunity in animal models is due to mainly CD8^+^ T cells and appears to target multiple antigens [11]. Several generations of vectored vaccines have now been assessed clinically in attempts to induce comparable efficacy [38,39]. However, even in mice, it has been difficult to generate high-level efficacy with vectors encoding single antigens, not least because the levels of T cells required are exceptionally high [40]. Currently, there are three approaches being assessed clinically.

The Oxford University programme, now partnered with the Italian biotech company Okairos, is using chimpanzee adenoviruses (ChAds) encoding the thrombospondin-related adhesion protein (TRAP) pre-erythrocytic antigen to prime an immune response [38] that is then boosted by another viral vector, modified vaccinia virus Ankara (MVA) that encodes the same TRAP insert [41]. This particular prime-boost approach, first discovered in malaria, leads to much higher T cell responses than single vector immunization, and extensive studies have shown its utility in pre-clinical models [42]. For example, Reyes-Sandoval *et al*. [41] showed enhanced efficacy against murine malaria using ChAds to prime and MVA to boost with not only increased efficacy but also better durability of protection. More recently, detailed analysis of this model has highlighted the importance for protection of effector CD8^+^ T cells that can reach the liver [43]. In the last year, very encouraging clinical efficacy results have been achieved using this approach in phase IIa sporozoite challenge trials, even against a parasite that encodes a different strain of *P. falciparum* TRAP [44]. This promising approach has now progressed to phase Ib safety and immunogenicity studies in African adults and children as part of the activities of a European and Developing Countries Clinical Trials Partnership (EDCTP-supported malaria-vectored vaccine consortium [45]. In an extension of this approach, further antigens are being tested in the same viral vectors including circumsporozoite protein (CSP) and the blood-stage antigens apical membrane antigen-1 (AMA1) and merozoite surface protein-1 (MSP1) (S. J. Draper, S. Sheehy & A. V. S. Hill 2010, unpublished data).

A related prime-boost approach is being developed by the US Naval Medical Research Centre, but here the priming vector is plasmid DNA, and a human adenovirus, Ad5, is used to boost the immune response [39]. As in the Oxford programme, several antigenic inserts from both pre-erythrocytic stage and blood stages are under assessment with some encouraging efficacy data in a recent challenge trial (T. Richie 2011, personal communication). Finally, the vaccine company Crucell has focused on using different adenoviral vectors such as Ad35 and Ad 26 [46] which, like chimpanzee vectors, may be less susceptible to impairment by naturally acquired immunity to common human adenoviral infections.

## 8. Blood-stage vaccines

In contrast to major progress in several areas of pre-erythrocytic vaccine development, results with blood-stage vaccines have been more mixed and progress has generally been slower [47]. A number of candidate vaccines have progressed to clinical testing but none has yet achieved good evidence of protective efficacy against clinical malaria. Many of these vaccine candidates are based on just a few antigens, MSP1 and AMA1 in particular, although there are hundreds or perhaps thousands of antigens expressed by blood-stage parasites that might be used in vaccine development. Almost all of these candidate vaccines have been a protein given with an adjuvant designed to induce protective antibodies. Some approaches have focused on inducing antibodies that impair parasite growth, as can be demonstrated in *in vitro* assays [48], whereas others have aimed to induce antibodies which achieve their effect in collaboration with effector cells and which can be measured, with greater difficulty, in a functional assay of ADCI of parasite growth [49].

There have been three particular challenges for the development of blood-stage vaccines. One is difficulty in expressing conformationally correct large antigens and scaling up the methods needed to do this to the extent that would allow large-scale manufacture. A second challenge has been the only modest antibody responses achieved even with a range of new adjuvants. In some cases, clear induction of antibodies with activity in impairing parasite growth in a standardized growth inhibitory assay has been achieved [48] but that has not been associated with significant vaccine efficacy. A telling comparator may be that strong adjuvants have generally induced antibody levels comparable to those seen in semi-immune adults living in endemic areas. Such adults have significant protective immunity from natural exposure because they have antibody responses to very large numbers of antigens not just one or two as in vaccinees. In contrast, antibody levels induced to CSP by RTS,S or T cells to TRAP induced by prime-boost vaccination are over a 100-fold higher than levels induced by natural exposure. The third difficulty has been posed by the extensive polymorphism of many leading candidate blood-stage antigens [50]: some vaccine candidates include more than one allele to try to overcome this but it remains to be seen how successful this approach will be.

A few hints of efficacy have been reported: switching of the parasite strain causing malaria away from the vaccine strain in a phase II trial of a blood-stage vaccine containing mono-allelic MSP2 in Papua New Guinea [51]; a possible reduction in parasite densities in a sporozoite challenge trial with AMA1/AS02 in US volunteers [52]; and one vaccinee showing a substantial delay in time to patency with the PEV3A virosomal vaccine candidate [53]. Vectored approaches to delivering blood-stage antigens are only beginning to be explored clinically and this approach should allow for evaluation of candidate antigens that have been impossible to express adequately as proteins. Perhaps, the best hope for a really effective blood-stage vaccine component may lie with identification of better target antigens to use in a vaccine: there are still many under-explored and unexplored candidates.

## 9. Mosquito stage vaccines

Few aspects of malaria vaccine development are more fascinating than the concept of vaccination to prevent transmission at the mosquito stage of infection. The idea of using gametocyte or sexual stage parasite antigens to immunize individuals who might derive no direct benefit but protect their neighbours from becoming infected [54] has been described as altruistic vaccination. But this is a misnomer as such vaccines would be deployed in such a manner that the whole community would benefit and hence the term ‘community vaccine’ is becoming more popular. Recently, the broader term ‘vaccines that interrupt malaria transmission’ (VIMTs) has been introduced as any pre-erythrocytic or blood-stage vaccine that was highly efficacious could have an effect on transmission [22].

The principle that immunization with gametocyte or ookinete antigens could reduce or ablate oocyst development in the mosquito has been established for decades [54]. Efforts have been made to standardize membrane-feeding assays that allow sera from vaccinated animals or humans to be evaluated for their ability to reduce or prevent transmission. Recently, this approach has been supplemented by the *in vivo* use of transgenic parasites to assess the efficacy of antibodies induced by *P. falciparum* and *P. vivax* antigens [55] in preventing transmission of rodent parasites. These systems provide a means of rapidly assessing the likely efficacy of sera from vaccinees participating in phase I trials of mosquito-stage vaccines, a major advantage of this approach to malaria vaccine development. Indeed, in the only reported clinical trial a sexual-stage, Pfs25-based*, P. falciparum* vaccine generated significant transmission-blocking activity in some vaccinees, even though the trial was not finished owing to safety issues with the protein–adjuvant formulation employed [56].

In view of the potential of this approach to the prevention of malaria, it is surprising that it has been so little supported until recently. The main hurdle has been the concern that deployment of such a transmission-blocking vaccine would prove impractical. Because adults and older children can transmit malaria as well as infants and young children, the target population has to be all residents of an area where transmission reduction is the aim. This type of mass vaccination has been undertaken on occasion for other diseases but may prove logistically challenging.

However, in the last few years, two developments have greatly increased interest in this type of vaccine. The first has been renewed interest in malaria elimination and eradication. This has focused attention on the potential value of a transmission-blocking mosquito-stage vaccine in the final stages of malaria eradication [22]. Although this is likely still several decades away, development of a licensable stand-alone mosquito-stage vaccine might require that time-frame. The second development has been the demonstration that antigens from the *Anopheles* midgut wall, particularly the aminopeptidase APN1 that appears to be a receptor for ookinetes, may act as suitable transmission-blocking vaccine components [57]. This true mosquito-stage vaccine (the term is generally used to encompass parasite sexual-stage antigens and mosquito antigens) has the particular advantage that it may be effective against more than one species of malaria, a feature unlikely to be shared by any other type of vaccine candidate. With this encouragement, there is renewed interest in development of several mosquito-stage vaccine candidates, although some of these may be developed as components of a multi-stage vaccine rather than as stand-alone candidates.

## 10. Combination vaccines

Many textbook descriptions of malaria vaccine development conclude with the suggestion that the first highly effective vaccines are likely to include antigens from more than one stage of the parasite's life cycle. This would appear a logical conclusion given the difficulty so far in developing highly effective vaccines based on any single life cycle stage. However, some practical considerations argue against multi-stage vaccines, particularly the associated increased manufacturing cost of a multi-stage vaccine comprising several components unless these can be encompassed by a single delivery technology, such as a poxviral vector [58,59].

However, there is an important argument for combining two particular types of subunit vaccine in the near-term to try and achieve higher level vaccine efficacy in humans [38]. As described above, the two subunit vaccine strategies that have achieved most efficacy in humans are a protein/adjuvant vaccine, specifically RTS,S, that induces antibodies that clear sporozoites before they can enter the liver, and vectored vaccines that clear infected liver cells. In a murine *Plasmodium berghei* model, analogous antibody-inducing and T cell-inducing vaccines each provided 30–35% sterile efficacy when administered alone. However, when administered as a simple mixture, the two vaccines provided 90 per cent sterile efficacy [60]. This more than additive efficacy of the combination is not difficult to understand: when anti-sporozoite antibodies substantially reduce the number of parasites that can enter the liver, it should be easier for the vector-induced T cells to clear the smaller number of remaining parasite-infected hepatocytes.

Interestingly, there are fairly good estimates of how effective RTS,S and leading vectored vaccines are at reducing the number of parasites reaching blood-stage infection (i.e. leaving the liver) in human sporozoite challenge studies [25]. RTS,S reduces parasite numbers by over 95 per cent and vectored vaccines by over 90 per cent. Viewed in this way, there appears to be a compelling case for testing combinations of leading sporozoite antibody-inducing and liver-stage T cell-inducing vaccines in humans in the near future. This could considerably accelerate the identification of a highly effective deployable malaria vaccine using components that are already in clinical development.

## 11. Prospects

In recent years, there has been progress in developing anti-parasite vaccines for humans. The first is almost certainly going to be a malaria vaccine and it is likely that a variety of different types of malaria vaccine will be licensed in the next 5–15 years. A Malaria Vaccine Technology Roadmap was published in 2006 after a series of consultations with many different groups of stakeholders [61]. Two clear targets were identified: a first generation vaccine with efficacy of at least 50 per cent lasting 1 year by 2015, and a second generation vaccine with efficacy of at least 80 per cent lasting 4 years by 2025. It now looks as if RTS,S/AS01 will come close to, or meet, the 2015 efficacy goal and a variety of approaches outlined above, particularly multi-component protein plus vector combinations, look as if they could make the more difficult 2025 efficacy goal achievable.

In addition, new efforts are being accelerated to create two further types of malaria vaccine. The first is a transmission-blocking vaccine useful for reducing transmission further in low transmission areas, particularly as part of efforts at malaria eradication [22]. This could be a stand-alone mosquito-stage vaccine or, more likely, a multi-component vaccine adding a mosquito-stage component to the partial transmission-blocking activity of a pre-erythrocytic vaccine. The second target, that is now receiving much overdue attention, is the development of a vaccine against *P. vivax* [62]. This is geographically the most widespread human malaria and the first efficacy studies of *P. vivax* vaccines were being undertaken in 2010 assessing a CSP-based candidate.

Development of an effective malaria vaccine has been a great challenge for medical science but findings and approaches pioneered in efforts to develop a malaria vaccine are proving useful in developing a whole range of vaccines against other difficult diseases. The story continues into the second decade of this millennium but that period will almost certainly see the licensure of at least one vaccine against this great scourge of humanity.
